# Single-Unit Comonomer Insertion Initiates Radical Polymerization of Thionolactone to Give Chemically and Thermally Degradable Polythioesters

**DOI:** 10.1021/acs.macromol.5c01418

**Published:** 2025-09-05

**Authors:** Touseef Kazmi, Kyle S. Hepburn, Qamar Nisa, Swarnali Neogi, Nathaniel M. Bingham, Peter J. Roth

**Affiliations:** School of Chemistry and Chemical Engineering, 3660 University of Surrey, Guildford, Surrey GU2 7XH, U.K.

## Abstract

Polythioesters are promising as sustainable materials with improved properties. But, despite the industrial importance of radical polymerization, polythioester homopolymers are not readily accessible through this method. Using dibenzo­[*c*,*e*]­oxepin-5­(7H)-thione (DOT) as a thionolactone monomer, this study shows that the attempted radical homopolymerization suffers from low (3–13%) conversion. Conversely, addition of diethyl vinylphosphonate (DEVP), a judiciously chosen comonomer, led to near-quantitative DOT conversion at low (<22%) DEVP incorporation. Thorough analysis of the products by ^1^H–^1^H COSY, ^1^H–^13^C HMBC, and ^1^H–^31^P HMBC NMR spectroscopy revealed that DEVP was inserted as single units and enabled the identification of DEVP repeat units sandwiched between two DOT groups (δ_P_ = 24.5 ppm) and those located at the α end group between an AIBN-derived fragment and a DOT repeat unit (δ_P_ = 23.7 ppm). The proportion of single DEVP repeat units at the α end group increased with a decreasing DEVP copolymerization feed, demonstrating the importance of the secondary DEVP-based radical in effectively initiating the polymerization of DOT. Polymers that carried, on average, less than one internal DEVP unit and consisted mostly of AIBN-DEVP-initiated DOT homopolymers were prepared by tuning the DEVP feed ratio. The polymers were fully degradable through aminolysis and by heating (in bulk) to 140 °C, with a higher DEVP content found to increase the formation of dibenzo­[*c*,*e*]­thiepin-5­(7H)-one (DTO) thiolactone product during thermal degradation.

## Graphical abstract

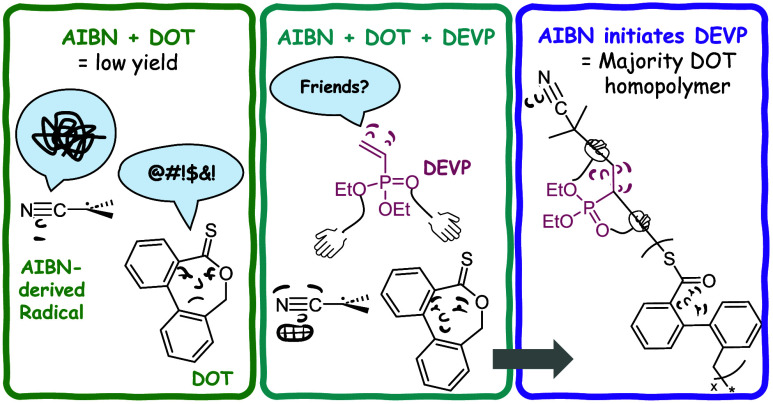

## Introduction

Polythioesters are receiving increasing attention from academia and industry as sustainable alternatives to classic poly­(oxo)­esters.^1–4^ The substitution of an oxygen for a sulfur atom has been shown to enhance mechanical, thermal, and optical properties^1–3^ and to facilitate degradation.^5,6^ Polythioesters are typically prepared through anionic/catalytic ring-opening polymerization (ROP) of thiolactones (cyclic —SC­(=O)—).^7–10^ Recent work also demonstrated the synthesis of thioesters through anionic^11^ and cationic^12,13^ ROP of thionolactones (cyclic —OC­(=S)—).^14^

Radical polymerization continues to be the main industrial plastic production process, with approximately half of the world’s plastics produced this way.^15,16^ The high functional group tolerance, high conversions under mild reaction conditions, applicability to various monomer classes, architectural design through reversible deactivation methods, and the wide scope of heterogeneous polymerization methods including ‘green’ conditions are main advantages of radical polymerization.^15,16^ But the literature on radically made thioester homopolymers is scarce.

Vinyl–thioester *co*polymers were first reported in 2019. Independently, our group^17^ and Gutekunst^18^ introduced the radical copolymerization of the thionolactone dibenzo­[*c*,*e*]­oxepin-5­(7H)-thione (DOT). Since then, other thionolactones have been reported and successfully copolymerized.^6,19–24^ The mechanism, termed thiocarbonyl addition–ring-opening (TARO)^17^ involves the reversible addition of a growing chain onto the C=S bond, followed by irreversible ring-opening, which produces a thioester and a benzylic radical that reinitiates the polymerization. Compared to the conventional radical ring-opening polymerization (RROP) of cyclic ketene acetals and cyclic allyl sulfides, the advantages of TARO are (i) stability of thionolactones at ambient conditions,^17^ (ii) rapid incorporation into acrylate-,^17,18^ acrylamide-,^25^ styrene-,^6,26^ maleimide-,^27^ isoprene-,^28^ *S*-vinyl-^29^ ethylene-,^30^ and vinyl ester^19,22^ -based copolymers, and (iii) selective degradation of thioesters in the presence of oxoesters through aminolysis,^17^ thiolysis,^31^ and oxidative hydrolysis.^25,31^ While the copolymerization of DOT and derivatives is promising for emerging applications in 3-D printing,^32^ recycling,^6^ biomedicine,^28,31^ as well as degradable nanoparticles,^26,33,34^ networks,^35^ and adhesives,^5^ the efficient radical homopolymerization of DOT has not been reported. The first studies showed that copolymerizations incurred retardation and low yields if the DOT feed exceeded 30–40 mol %.^17,18^ In the same vein, an AIBN-initiated homopolymerization of DOT gave <10% conversion. After repeated heating with subsequent additions of fresh AIBN, sufficient material was eventually isolated to allow for full NMR characterization.^27^ Surprisingly, a subsequent study found evidence of DOT–DOT diads forming during acrylamide copolymerizations, indicating that DOT self-propagation is a competitive process in copolymerizations.^25^ The homopolymerization of a small number of other cyclic thiocarbonyl monomers has been attempted. ε-Thionocaprolactone was reported to form a homopolymer in the presence of 50 mol % of a cyclic ketene acetal comonomer,^22^ whereas other cyclic thiocarbonyl monomers underwent slow^23,24^ homopolymerization, required elevated temperature,^21^ or showed no homopolymerization at all.^19,36^ Given the increasing relevance of polythioesters as materials and the industrial prevalence of radical polymerization methods, it is important to explore efficient radical homopolymerization of thionolactones.

In this work, we detail a series of unsuccessful experiments to prepare DOT homopolymers under varying polymerization conditions. Hypothesizing that the reversible addition equilibrium of AIBN lay on the left and prevented effective initiation, we sought a poorly reactive comonomer to facilitate initiation. Gratifyingly, we found that diethyl vinylphosphonate (DEVP) fulfilled this role well: In a series of copolymerizations with increasing DEVP feed, 10 mol % DEVP (relative to the DOT feed) was sufficient to increase the DOT conversion to 74%, while up to quantitative DOT conversion was found for higher DEVP feeds. DEVP, on the other hand, showed low conversions (5–22%). The high sensitivity of the ^31^P nuclei to their chemical environment was leveraged in ^1^H–^31^P HMBC NMR spectra that showed that, with a decreasing DEVP feed, an increasing percentage of single DEVP repeat units was located at the α end group of the chains, demonstrating the important role of the secondary radical on DEVP to initiate the polymerization of DOT. For low DEVP feeds, most chains were found not to contain any internal DEVP repeat units, demonstrating efficient DOT homopolymerization in the presence of an initiating comonomer.

## Experimental Section

### Chemicals

Dibenzo­[*c*,*e*]­oxepin-5­(7H)-thione (DOT)^17^ and poly­[oligo­(ethylene glycol) methyl ether acrylate] macro-RAFT agents (PEGA_24_ *M*_n_ = 6.4 kg/mol, *Đ* = 1.20 and PEGA_99_ *M*_n_ = 9.7 kg/mol, *Đ* = 1.13)^31^ were prepared as described elsewhere. All other reagents were purchased from Sigma-Aldrich and used as received unless noted otherwise. Ethylamine (2 M in THF) was purchased from Thermo Scientific and diluted with THF (1:1) prior to the aminolysis of polymers. Vinyl monomers were deinhibited by passing through a plug of basic alumina to remove inhibitors immediately before polymerization. 2,2′-Azobis­(isobutyronitrile) (AIBN) was recrystallized from methanol and stored at −20 °C.

### Instrumentation

NMR spectra were recorded on a 400 or 500 MHz Bruker spectrometer in CDCl_3_ with the residual solvent signal of CHCl_3_ used as reference (δ = 7.26 ppm).

Fourier transform infrared (FT-IR) spectroscopy was performed by using a PerkinElmer FT-IR spectrometer with attenuated total reflection (ATR).

Size exclusion chromatography (SEC) was performed on a Viscotek GPCMax VE 2001 setup with three linear 7.5 mm × 300 mm PLgel mixed columns connected to a Viscotek VE3580 refractive index (RI) detector. The instrument operated at 35 °C with tetrahydrofuran (THF) containing 250 ppm of BHT, as mobile phase at a flow rate of 1.0 mL/min. The calibration of the system was based on the relative molar mass determination of a series of narrow molecular weight distribution poly­(methyl methacrylate) (pMMA) standards ranging from 0.88 to 1677 kg/mol, and the reported values are pMMA equivalent.

Thermogravimetric analysis (TGA) was conducted using a TGA Q500 (TA Instruments, New Castle, New Jersey). A sample weighing 2–5 mg was placed in a platinum pan and subjected to heating at a rate of 10 °C/min from room temperature to 800 °C under a nitrogen atmosphere, with a flow rate of 60 mL/min.

Differential scanning calorimetry (DSC) was performed on a DSC Q1000 (TA Instruments, New Castle), calibrated with indium. A sample of approximately 2–5 mg was weighed and sealed in an aluminum hermetic pan, with an empty pan serving as the reference. The glass transition temperature (*T*_g_) was measured using a heat–cool–heat cycle from −60 to 210 °C under a nitrogen atmosphere, with heating and cooling rates of 10 °C/min. The midpoint *T*_g_ was reported from the second heating cycle.

### General Procedure for Polymerization

Typically, DOT (68.0 mg, 0.3 mmol, 50 equiv), the comonomer or additive (in varying molar ratios as described in the main text), AIBN (1 mg, 6 μmol, 1 equiv), and solvent anisole (2 mL) were added into a round-bottom flask. The mixture was sealed with a rubber septum and degassed with nitrogen for 30 min through a needle with a shorter needle fitted for gas release. The tube was heated in an oil bath at 70 °C for the durations described in the main text. After cooling and exposure to air, the monomer conversion was determined by ^1^H NMR spectroscopy of a sample of the crude mixture diluted with CDCl_3_. Residual monomer signals (DOT methylene group at 5.2 ppm and DEVP vinyl signals) were compared with signals of the polymer (1 H of DOT repeat unit at δ_H_ = 6.98 ppm and DEVP backbone methylene at 1.8–2.8 ppm). Polymers were purified by precipitation into a 40-fold excess of methanol or diethyl ether–hexane 1:1, followed by centrifugation, decanting, and drying *in vacuo*.

### RAFT Copolymerization

A RAFT-mediated polymerization was conducted as described above using a mixture of DOT (68.0 mg, 0.3 mmol, 50 equiv), DEVP (24.6 mg, 0.15 mmol, 25 equiv), RAFT agent *S*-benzyl-*S*′-propyl trithiocarbonate (1.45 mg, 6 μmol, 1 equiv), AIBN (0.25 mg, 1.5 μmol, 0.25 equiv), and solvent anisole (2 mL).

### Copolymerization Kinetics

The copolymerization kinetics were recorded by using a DOT–DEVP–AIBN ratio of 50:50:1. Briefly, DOT (68.0 mg, 0.3 mmol, 50 equiv), DEVP (49.2 mg, 0.3 mmol, 50 equiv), AIBN (1 mg, 6 μmol, 1 equiv), internal standard naphthalene (20.0 mg, 0.156 mmol, 25.6 equiv), and solvent anisole (3 mL) were combined in a tube and reacted as described above. Aliquots of 0.2 mL were withdrawn from the reaction mixture at intervals from 20 min to 24 h under nitrogen counterflow and diluted with CDCl_3_ for ^1^H NMR analysis. The comonomer conversions of aliquots were determined by comparison of the residual ^1^H monomer signals with those of the internal standard and with a ^1^H NMR spectrum recorded prepolymerization.

### Aminolysis of Copolymers

(Co)­polymer (1–5 mg) was dissolved in ethylamine (1 M in THF, 2 mL), and the mixture was stirred overnight at RT. The solvent was then evaporated by using a stream of nitrogen. The dry residue was redissolved in THF for SEC, or in CDCl_3_ for analysis by ^1^H NMR spectroscopy.

### Thermal Degradation

Copolymers were placed in test tubes (in the absence of solvent or inert atmosphere), closed loosely with Al foil, and immersed in a preheated oil bath set to 140 °C. Aliquots were withdrawn at intervals to monitor the degradation over a period of 30 days. The degraded copolymer samples were dissolved in THF for SEC analysis or in CDCl_3_ for ^1^H NMR spectroscopy. The percentage of formed dibenzo­[*c*,*e*]­thiepin-5­(7H)-one (DTO) was determined by comparing the methylene signals of DTO (two doublets at δ_H_ = 4.29 and 3.58 ppm) with all aromatic signals (excluding the residual solvent signal).

## Results and Discussion

### DOT Homopolymerization Attempts

Since our first report of DOT (co)­polymerization in 2019, we have attempted to homopolymerize DOT under a range of conditions; see Scheme 1A and Table S1. Typical polymerizations involved AIBN (3 mM) and DOT (0.15 M, [DOT]_0_/[AIBN]_0_ = 50), while others used less AIBN (1.5 mM, [DOT]_0_/[AIBN]_0_ = 100) with heating overnight to 70 °C. A range of solvents (acetonitrile, acetonitrile-*d*_3_, DMSO, DMSO-*d*_6_, DMF, acetic acid, ethyl acetate, toluene-*d*_8_, and anisole) were used with DOT conversions generally between 2 and 13 mol % (Table S1, entries 1–13). An exception was a single formulation in toluene that gave 31 mol % conversion, and a monomodal SEC-measured curve with *M*_n_ = 12.2 kg/mol and *Đ* = 2.18 (entry 8); see Figure 1A. A repeat experiment, however, only gave 12 mol % conversion (entry 9). Mixtures of toluene–octane and toluene–ethanol (entries 11 and 12) gave similarly low conversions. A polymer prepared in anisole with 13 mol % conversion showed a very similar monomodal SEC curve as the toluene-prepared sample, with a measured *M*_n_ = 12.0 kg/mol and *Đ* = 2.05, Figure 1B. Anisole was chosen as a solvent to explore further conditions.

**Figure 1:**
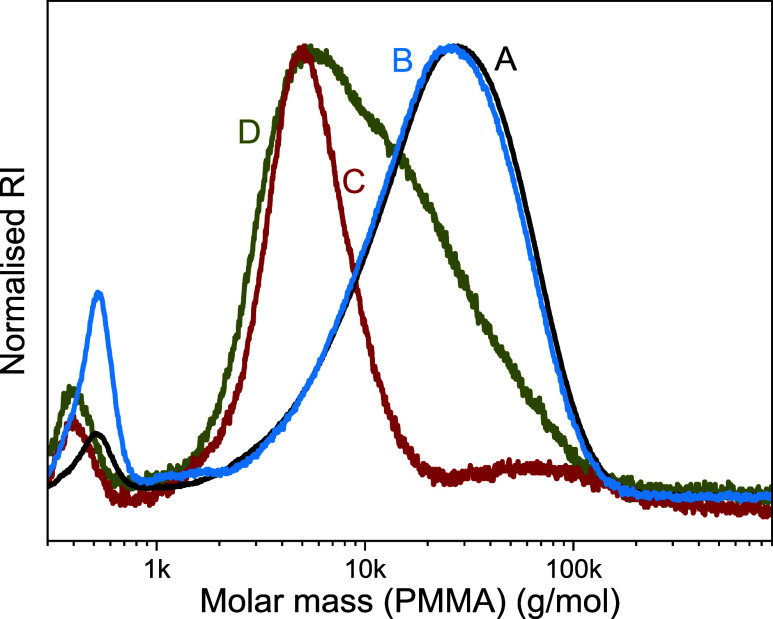
SEC data of DOT homopolymers prepared through (low-yielding) free radical homopolymerization: AIBN-initiated polymerization of DOT (0.15 M) in (A) toluene (Table S1, entry 8) and (B) anisole (entry 13); (C) AIBN-initiated polymerization of DOT (0.6 M) in anisole (entry 14); and (D) di-*tert*-butylperoxide-initiated polymerization of DOT (0.15 M) in anisole at 120 °C (entry 18).

**Scheme 1:**
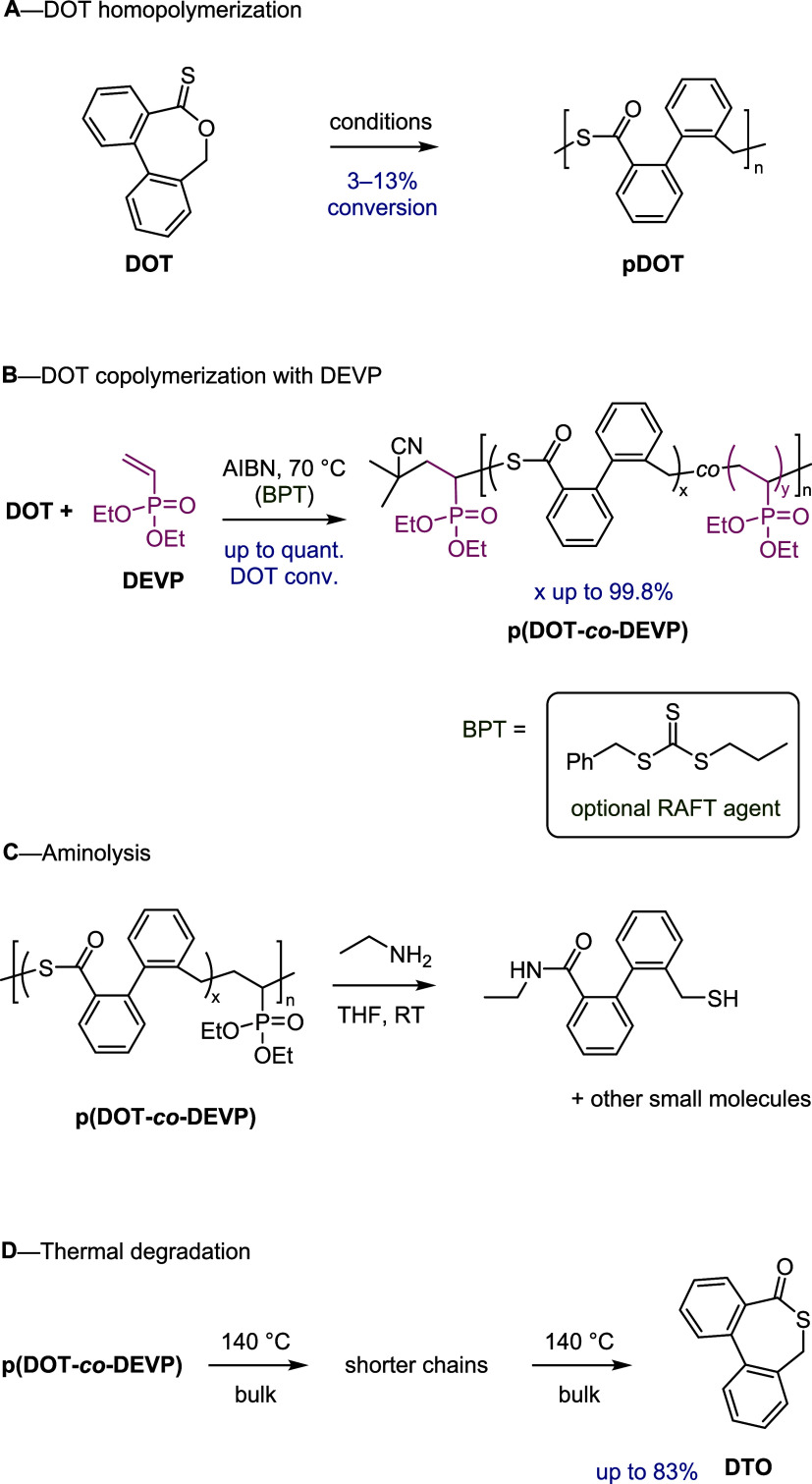
(A) DOT Homopolymerization; (B) DOT Copolymerization with Diethyl Vinylphosphonate (DEVP) with Structure of RAFT Agent *S*-Benzyl-*S*′-propyl Trithiocarbonate; (C) Aminolysis to Small-Molecule Fragments; and (D) Thermal Degradation (in the Absence of Solvent) Showing Cleavage of Thioesters Followed by Cyclization to Give the DOT Isomer DTO

Notably, the addition step of radicals onto DOT is expected to be reversible.^37^ As detailed by Harrisson et al.,^38^ in this scenario, the initial concentration of the cyclic monomer (and its influence on the position of the addition equilibrium) can have a strong impact on the polymerization efficiency. However, our polymerization attempt using a four times higher concentration of DOT (0.6 M) in anisole (Table S1, entry 14) only gave 3% DOT conversion. The polymer had a lower measured molar mass, *M*_n_ = 4.7 kg/mol compared to the sister species made at lower DOT concentration and had a markedly lower dispersity (*Đ* = 1.30), Figure 1C, suggesting perhaps the storage of radicals through reversible addition similar to thiocarbonyl spin traps.^39^ Due to the generally poor solubility of DOT in organic solvents, higher concentrations were not tested.

Prebihalo et al.^23^ described the unusually slow homopolymerization of thionoisochromanone. The surprisingly low dispersities (as low as *Đ* = 1.12) resulted from a sustained orthoester chain end formed from the attack of the (carbon-based) intermediate radical on another thiocarbonyl monomer. High conversions were achieved by increasing the polymerization time to up to 8 days. In the current case of DOT, however, increasing the polymerization time of an anisole-based formulation to 1 week gave 6 mol % conversion of DOT (Table S1, entry 15), suggesting the absence of the radical storage effect found for thionoisochromanone.

Plummer et al.^22^ demonstrated that, when using AIBN as initiator, ε-thionocaprolactone homopolymerized in the presence of the cyclic ketene acetal (CKA) 2-methylene-1,3-dioxepane, but not by itself. The authors speculated that the more reactive CKA-based radical was necessary to initiate the homopolymerization of ε-thionocaprolactone. Using computational modeling, Luzel et al.^21^ showed that the bottleneck for DOT polymerization is the ring-opening step. Slow ring-opening (fragmentation) may lead to retardation.^40^ Given the entropic gain achieved by ring-opening, this step is usually more efficient at elevated temperatures. We therefore trialed the homopolymerization of DOT using lauroyl peroxide, benzoyl peroxide, and di-*tert*-butylperoxide at temperatures up to 120 °C. Unfortunately, however, DOT conversions did not exceed 3% in these experiments (Table S1, entries 16–19). SEC analysis of a di-*tert*-butylperoxide-initiated polymer with 3% DOT conversion showed a bimodal curve with *M*_n_ = 6.8 kg/mol and *Đ* = 2.31, Figure 1D. As a reviewer pointed out, given the limited number of rotatable bonds generated upon ring-opening DOT, the entropic gain may not be as large as that for other cyclic monomers.

Since in copolymerizations, DOT self-propagation has been observed through the formation of DOT–DOT diads,^25^ we speculated about a molecular weight-dependent retardation effect^41^ which could be leading to termination of oligomeric DOT-centered radicals, thus preventing the homopolymerization but not the self-propagation at the end of an established chain. We therefore attempted to use RAFT-made poly­[oligo­(ethylene glycol) methyl ether acrylate] macro-RAFT agents to initiate DOT in the presence of polymer-based radicals but with, unfortunately, no improvement in conversion (Table S1, entries 20–21).

In the context of exploring the influence of different solvents on the reactivity of DOT, we ran ^1^H NMR spectra of DOT in various (deuterated) solvents. Interestingly, the signals attributed to the CH_2_ group varied significantly between solvents, with some (anisole, toluene–hexane 1:1, toluene–octane 1:1) giving singlets, while other solvents (CDCl_3_, DMSO-*d*_6_, acetonitrile-*d*_3_, acetic acid, dioxane, DMF, ethyl acetate, methyl acrylate, and THF) showed the characteristic two doublets. Variable temperature ^1^H NMR spectroscopic measurements run in DMSO-*d*_6_ and toluene-*d*_8_ confirmed that the two doublets converged into a singlet when heated and that the thermal barrier for the inversion of the seven-membered ring varies between solvents; see Table S2, Figures S1 and S2. However, no correlation between the appearance of the ^1^H NMR spectra and the conversions in the same solvents was found.

### Copolymerization of DOT with Diethyl Vinylphosphonate (DEVP)

Based on reported DOT–DOT diads formed in copolymerizations, we considered the use of a vinyl comonomer to form DOT-rich copolymers. Previously, we explored the copolymerization of DOT with a selection of S-vinyl and P-vinyl monomers including a single example of a copolymerization of DOT with diethyl vinylphosphonate (DEVP) that led to a DOT-rich copolymer.^29^ Herein, the case of DEVP is explored further, Scheme 1B. This comonomer is, perhaps, unique for two reasons: (i) DEVP has been reported to poorly homopolymerize to high molar masses, making it ideal to favor crosspropagation and (ii) the AIBN-initiated homopolymerization of DEVP has been shown to proceed via chain transfer to monomer, which likely involves a higher-energy radical.^42^

Copolymers were made under similar conditions as above using anisole as the solvent with [DOT]_0_ = 0.15 M and [AIBN]_0_ = 3 mM but with varying amounts of added DEVP. All copolymerizations are summarized in Table 1. The first experiment used a feed ratio of 50 DOT and 50 DEVP (relative to 1 AIBN) and, surprisingly, led to near-quantitative DOT conversion with a 22% conversion of the DEVP feed to give a final 82 mol % DOT content in the copolymer (Table 1, entry 1). Next, a series of copolymers was prepared with decreasing DEVP feed (of 25, 10, 5, 3, 2, 1 equiv) to investigate the minimum DEVP feed needed to achieve a high DOT conversion, Table 1, entries 2–7. The results, plotted in Figure 2, showed that the DEVP conversion was low (1117%) for all experiments with a gentle increase with a higher feed. The conversion of DOT, however, was greatly affected by the DEVP additive, increasing from 13% (for no added DEVP) to (near-)­quantitative (for 25 and 50 equiv of added DEVP). Due to higher feed and slightly higher conversion of DEVP, the DOT content in the copolymers decreased with increasing DEVP feed from 100% (no DEVP additive) to 82% (50 equiv of DEVP). A DEVP feed of 5–10 equiv was considered ideal because it led to high (74 and 87%) DOT conversion and high DOT content of 98–99%; see Figure 2. ^1^H NMR spectroscopy confirmed the incorporation of DEVP repeat units showing multiple DEVP backbone signals (with splitting due to ^1^H–^31^P coupling), as well as ethyl signals (with the methylene group signals overlapping with the methylene group signal of DOT–DOT diads); see Figure 3. Copolymerization kinetics was done on a polymerization with a 50 DOT–50 DEVP feed. To allow withdrawing multiple samples from a master batch, more solvent was used, which led to lower global conversion, but the data, shown in Figure 4A, clearly demonstrated the increasing consumption of DOT with time. The DEVP consumption, while overall very low, was faster at the beginning of the polymerization leading to a slightly higher (10–20 mol %) DEVP content during the first 3 h of the copolymerization with a cumulative DEVP content of about 6 mol % reached after 24 h of polymerization. SEC traces (Figure 4B) run of the withdrawn aliquots showed nearly identical distributions as expected of radical polymerization. Reactivity ratios for the DOT–DEVP pair were estimated from this copolymerization experiment by fitting the conversion data with a numerical solution of the Mayo–Lewis equation to be *r*_DOT_ = 17 and *r*_DEVP_ = 0.01; see Figure S3 and accompanying details. Being based on a single copolymerization experiment, these values are rough estimates only but nonetheless demonstrate the stark difference in the reactivity between the two monomers.

**Figure 2:**
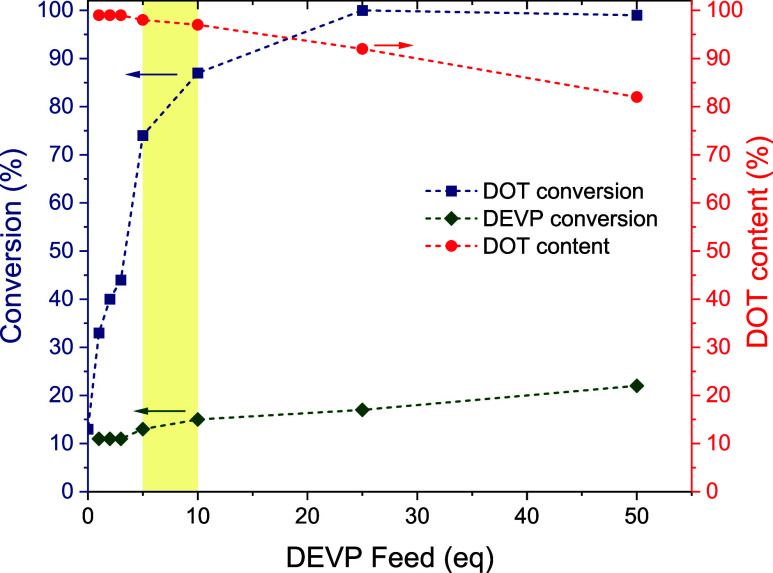
Conversion (left axis) of DOT (blue squares) and DEVP (green diamonds) and copolymer DOT content (estimated from conversions) (red circles, right axis) versus DEVP feed for a series of copolymerizations containing a feed of 50 DOT and 1 AIBN (Table S1, entry 13, and Table 1, entries 1–7). Dashed lines were added to guide the eye. A “sweet spot” with high (74–87%) DOT conversion and near-quantitative (98–99%) copolymer DOT content is highlighted by the yellow band.

**Figure 3:**
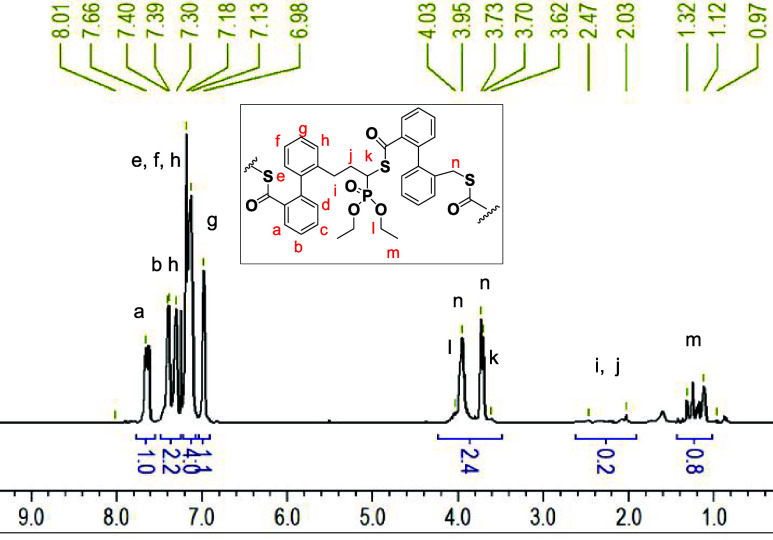
^1^H NMR spectrum of p­(DOT_0.92_-*co*-DEVP_0.08_)*_n_* (Table 1, entry 2) with assignments.

**Figure 4:**
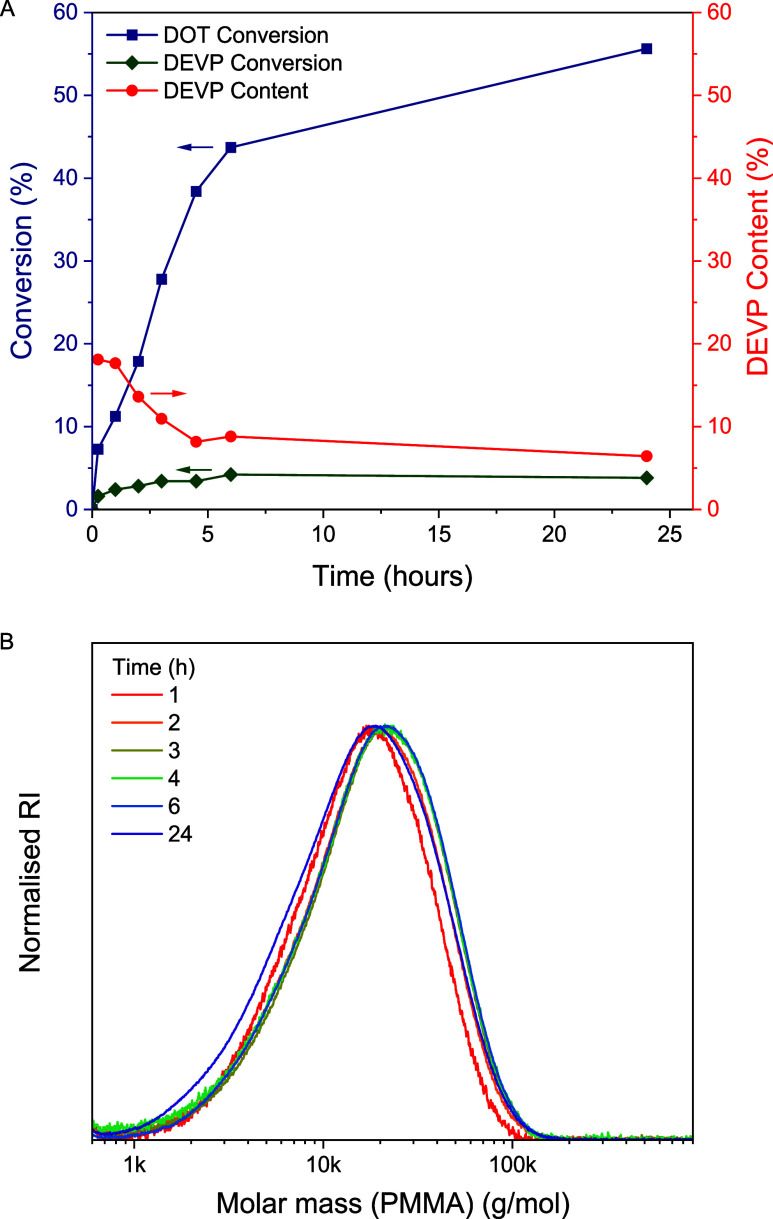
Kinetics of a 50 DEVP–50 DOT copolymerization (Table 1, entry 8): (A) Conversion (left axis) of DOT (blue squares) and DEVP (green diamonds) and cumulative DEVP content (red circles, right axis) and (B) SEC curves of the aliquots.

**Table 1:** Polymerization Data for p­(DOT-*co*-DEVP)*_n_* ^a^

entry	code	feed DOT; DEVP (equiv)^b^	conversion DOT; DEVP (%)^c^	DOT content (mol %)^c^	*M*_n_^SEC^ (kg/mol)	*Đ*_M_^SEC^	*T*_g_ (°C)
1	p(DOT_0.82_ *-co*-DEVP_0.18_)*_n_*	50; 50	99; 22	82	15.9	1.98	
2	p(DOT_0.92_-*co*-DEVP_0.08_)*_n_*	50; 25	100; 17	92	19.3	1.74	95.3
3	p(DOT_0.97_-*co*-DEVP_0.03_)*_n_*	50; 10	87; 15	97	15.4	1.75	
4	p(DOT_0.98_-*co*-DEVP_0.02_)*_n_*	50; 5	74; 13	98	19.0	1.94	102.7
5	p(DOT_0.985_-*co*-DEVP_0.015_)*_n_*	50; 3	44; 11	98.5	17.7	2.04	
6	p(DOT_0.99_ *-co*-DEVP_0.01_)*_n_*	50; 2	40; 11	99	24.8	2.07	104.0
7	p(DOT_0.994_-*co*-DEVP_0.006_)*_n_*	50; 1	33; 11	99.4	25.2	1.86	
8	p(DOT_0.94_ *-co*-DEVP_0.06_)*_n_*	50; 50	56; 4	94	10.3	2.13	
9	p(DOT_0.99_ *-co*-DEVP_0.01_)*_n_*	100; 5	25; <5	99	23.8	2.10	
10	p(DOT_0.994_-*co*-DEVP_0.006_)*_n_*	200; 5	25; 6	99.4	40.6	1.79	
11^d^	p(DOT_0.93_ *-co*-DEVP_0.07_)	50; 25	94; 15	93	5.1	1.19	
12	pDEVP	0; 100	0; 82	-	13.1	2.20	

a All polymerizations used AIBN (1 equiv) and heating to 70 °C overnight.

b Relative to 1 equiv of AIBN.

c Determined by integration of ^1^H NMR spectrum of the polymerization mixture before workup.

d RAFT agent *S*-benzyl-*S*′-propyl trithiocarbonate (1 equiv) used together with 0.25 equiv AIBN.

FT-IR spectroscopy run on a DOT homopolymer and DOT–DEVP copolymers (containing 2 and 8 mol % DEVP) showed virtually identical spectra with no discernible bands associated with the DEVP repeat units, in agreement with the high DOT content observed by ^1^H NMR spectroscopy, Figure S4. Differential scanning calorimetry run on the same samples showed a reduction of the measured glass transition temperatures of 105 °C for the DOT homopolymer with increasing DEVP content, Figure S5. This observation is to be expected from the incorporation of DEVP repeat units based on the published *T*_g_ of DEVP homopolymer of −62 °C.^43^ Thermal gravimetric analysis (TGA) run on a DOT homopolymer and DOT–DEVP copolymers (containing 1, 2, and 8 mol % DEVP) under a nitrogen atmosphere showed thermal stability of all samples up to 300 °C with no influence of the DEVP repeat units, Figure S6. SEC analysis of the DOT–DEVP copolymers gave dispersities between 1.74 < *Đ* < 2.13, suggesting the absence of side reactions that influence the molecular weight distribution. Further copolymers were prepared by keeping the [DEVP]_0_:[AIBN]_0_ ratio constant at 5:1 but with higher [DOT]_0_ values of 100 and 200 (Table 1, entries 9 and 10). The DOT conversions for these cases were low (around 25%) but, expectedly for the higher monomer feed, polymers with higher molar mass were obtained; see Figure 5, demonstrating control over the resulting degree of polymerization. A further copolymerization using [DOT]_0_:[DEVP]_0_ = 50:25 was done in the presence of 1 equiv of *S*-benzyl-*S*′-propyl trithiocarbonate as a RAFT agent (Table 1, entry 11). The comonomer conversions were similar to those of the sample prepared in the absence of the RAFT agent (Table 1, entry 2), leading to a similar overall DOT content of 93%. But, gratifyingly, the RAFT-made sample showed a low SEC-measured dispersity of *Đ* = 1.19, indicating the successful and high-yielding preparation of a DOT-rich copolymer with low dispersity.

**Figure 5:**
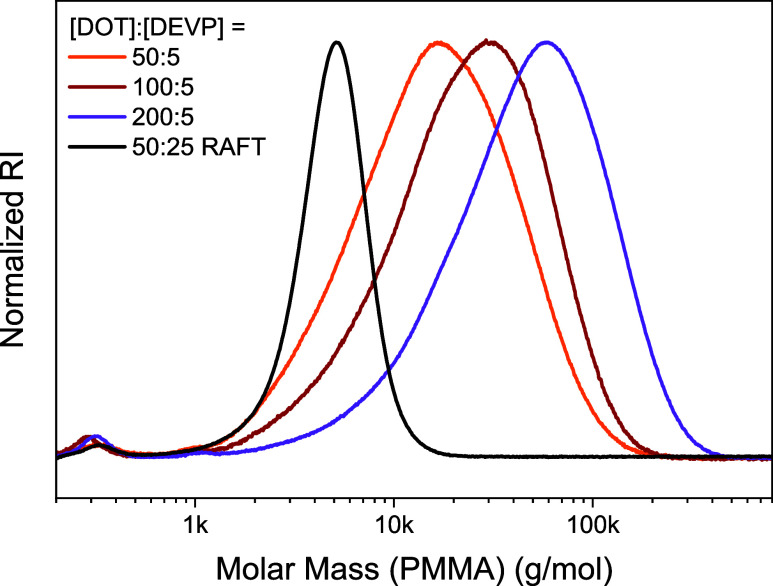
SEC curves of DOT–DEVP copolymers prepared by radical polymerization with [DOT]_0_:[DEVP]_0_ = 50:5 (orange curve, Table 1, entry 4), 100:5 (red curve, Table 1, entry 9), and 200:5 (purple curve, Table 1, entry 10), as well as a RAFT-mediated copolymerization with [DOT]_0_:[DEVP]_0_ = 50:25 (black curve, Table 1, entry 11).

### Role of DEVP in Promoting “Homo”Polymerization of DOT

We next sought to understand the role of DEVP in the “homo”polymerization of DOT. DEVP has been shown to undergo chain transfer to a monomer through abstraction of a hydrogen atom from an OCH_2_ group. Reinitiation from this position leads to a (degradable) phosphonate in the backbone.^44^ Chain transfer to the monomer is expected to decrease the molecular weight with an increasing feed of that monomer. Indeed, for the series of copolymers prepared with [DOT]_0_ = 50 equiv (Table 1, entries 1–7), the SEC-measured molecular weights decreased from 25.2 to 15.4 kg/mol as [DEVP]_0_ increased from 1 to 10 equiv. With higher feeds, however, the SEC-measured molecular weights remained similar. A Mayo plot of the first five values of this series (Figure S7) gave a chain transfer agent of *c* = 0.03, suggesting a minor presence of chain transfer events to the monomer, at least at low DEVP feeds.

To gain further insight into the role of DEVP, the copolymers were analyzed by ^31^P NMR spectroscopy. As an example, the spectrum of p­(DOT_0.92_-*co*-DEVP_0.08_)*_n_* (Table 1, entry 2) is shown in Figure 6 (top spectrum). Notably, the spectrum showed two major sets of signals: an apparent triplet at δ_P_ = 24.5 ppm and an apparent doublet at δ_P_ = 23.7 ppm, with a further minor signal at δ_P_ = 22.9 ppm. These shifts were similar to the value of δ_P_ = 23.3 ppm published for a comparable small molecule featuring a phosphonate adjacent to a thioester, (EtO)_2_ P­(=O)–CH­(Me)­(SAc).^45^ Recording the same spectrum on a 400 MHz and a 500 MHz spectrometer (the frequencies for ^31^P being 162 and 202 MHz, respectively), indicated that the apparent triplet and doublet splitting were not due to coupling (which would have given the same coupling constants) but to different environments (with both spectra giving the same chemical shifts); see Figure S8 and Table S3. We assumed the splitting to be due to triad^46^ tacticity, with the apparent triplet signals presumably arising from a more complex environment than the apparent doublet.

**Figure 6:**
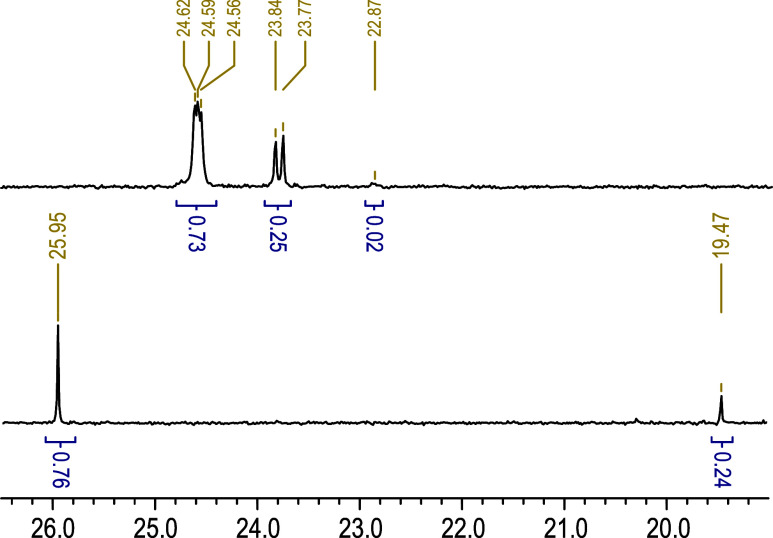
Stacked ^31^P {^1^H} NMR spectra of intact (top) and ethylamine-degraded (bottom) copolymer p­(DOT_0.92_-*co*-DEVP_0.08_)_n_ (Table 1, entry 2).

As a comparator, a DEVP homopolymer (Table 1, entry 12) was prepared. ^31^P NMR analysis (Figure S9) showed signals around δ_P_ = 29–33 ppm. Little difference was found between ^31^P and ^31^P {^1^H} spectra, supporting the notion that the splitting was due to different phosphorus environments and not coupling to hydrogen nuclei. Notably, the DEVP homopolymer signals were different from those of the DEVP–DOT copolymers, suggesting the absence of DEVP–DEVP diads in the copolymers. Further support for this insight came from the observation that all of the ^31^P copolymer signals shifted upon aminolysis with ethylamine (1 M in THF) of the DOT thioesters. The spectrum (Figure 6, bottom) showed sharp signals at δ_P_ = 26.0 and 19.5 ppm. A further minor (4%) signal was observed at δ_P_ = – 20 ppm, Figure S10. When the DEVP homopolymer was treated similarly with ethylamine, no change was observed apart from a similar minor (0.1%) signal at δ_P_ = −20 ppm, which was attributed to substitution of an ethoxy group with ethylamine on phosphorus, Figure S11. The stark changes of the copolymer ^31^P NMR spectra upon aminolysis (Figure 6) thus suggested that each DEVP repeat unit was followed by a DOT comonomer, in agreement with the literature chemical shift of an adjacent thioester (see above). The two distinct phosphorus environments (apparent triplet and apparent doublet) were assumed to originate from different preceding repeat units. To understand the spectra better, a model system was prepared by reacting a 1:1:1 mixture of DOT, DEVP, and AIBN (under otherwise unchanged polymerization conditions) to produce oligomers. This model showed the same apparent doublet at δ_P_ = 23.7 ppm and apparent triplet and δ_P_ = 24.5 ppm. Analysis by ^1^H–^31^P HMBC NMR spectroscopy gave the ^1^H chemical shifts of all hydrogens coupling with the two ^31^P environments, which were fully assigned with the aid of ^1^H–^1^H COSY and ^1^H–^13^C HMBC NMR spectroscopy, Figure 7. The two phosphorus types had different environments including different ^1^H and ^13^C shifts of the ethyl groups. The chemical shifts assigned to the DEVP PCH methine hydrogens, δ_H_ = 3.88 and 3.64 ppm, respectively, were similar to the chemical shift of δ_H_ = 3.84 ppm reported for (EtO)_2_P­(=O)–CH­(Me)­(SAc).^45^ Most significantly, the phosphorus signal at δ_P_ = 23.7 ppm was found to couple through five bonds (a distance known and exploited in the literature)^47^ with a singlet hydrogen environment at 1.33 ppm, which, in turn, coupled to carbon atoms at δ_C_ = 26, 31, and 124 ppm, characteristic of an AIBN fragment with its unique nitrile ^13^C chemical shift at 124 ppm, Figure S12. This phosphorus signal was thus attributed to a DEVP group sitting at the α end group of a chain, Figure 7 (top). The apparent triplet at δ_P_ = 24.5 ppm, on the other hand, was assigned to a DEVP repeat unit sandwiched between two DOT repeat units, supported by the coupling of the phosphorus signal to protons at δ_H_ = 2.05 ppm, which, in turn, coupled to three aromatic DOT carbons near the benzylic position; see Figure 7(bottom) and Figure S12. Coupling of the DEVP PCH methine hydrogens with the DOT thioesters (δ_C_ = 192 ppm) was, unfortunately, not visible due to overlap with the benzylic positions of DOT–DOT diads at δ_H_ = 3.96 and 3.71 ppm, which coupled to the thioester signal (δ_C_ = 192 ppm) (see Figure 3).

**Figure 7:**
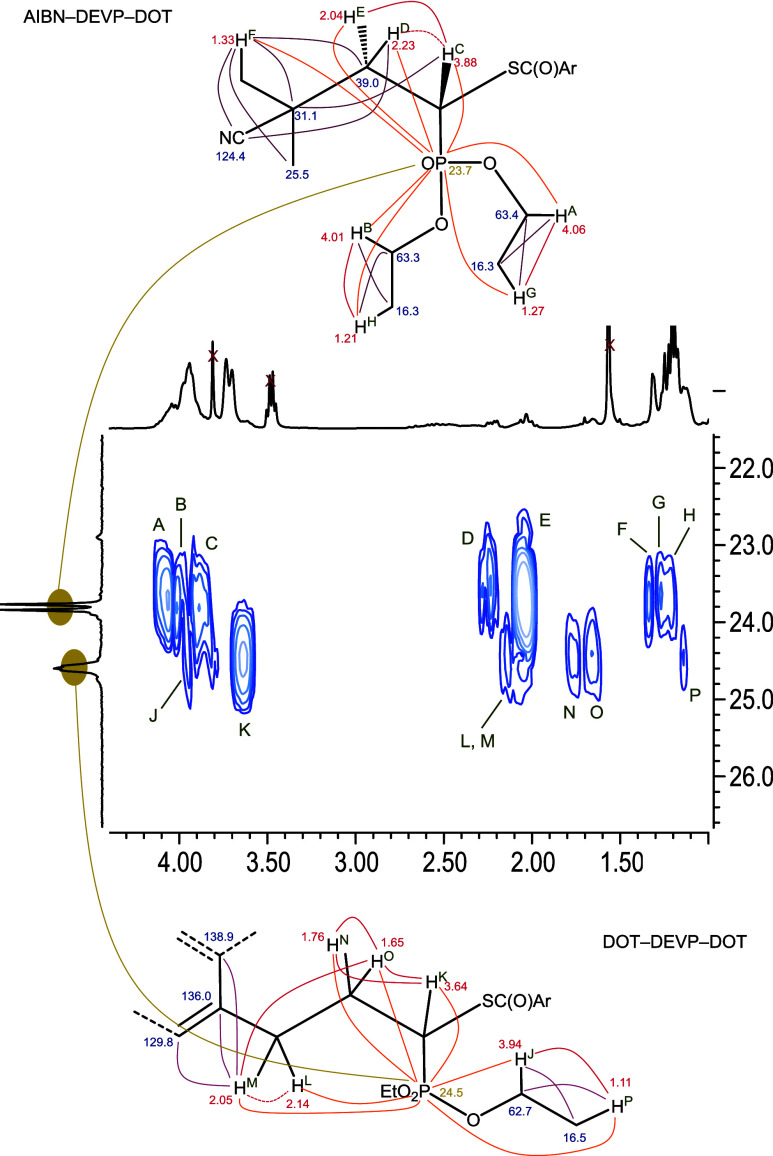
^1^H–^31^P HMBC spectrum (middle) with structures of two distinct phosphorus environments. Notably, this 2-D spectrum shows hydrogen atoms that couple with phosphorus, while the 1-D spectrum at the top shows all hydrogen atoms in the sample. Numbers in the structures indicate the observed chemical shifts (ppm) for ^1^H (red), ^13^C (blue), and ^31^P (yellow) nuclei; lines indicate coupling observed by ^1^H–^1^H COSY (red), ^1^H–^13^C HMBC (purple), and ^1^H–^31^P HMBC (orange) NMR spectroscopy. Dashed lines indicate weaker interactions which were interpreted to reflect different dihedral (Karplus) angles.

After interpreting the major signals in the ^31^P {^1^H} NMR spectra of the model compound, we compared the spectra of the series of DOT–DEVP copolymers, as shown in Figure 8. The spectrum of the copolymer made with the highest DEVP feed, p­(DOT_0.82_-*co*-DEVP_0.18_)*_n_* (Table 1, entry 1), showed a majority (84 mol %) of internal DEVP units sandwiched between DOT repeat units (Figure 8, spectrum A). But when the DEVP feed was reduced, the percentage of DEVP units located next to AIBN fragments increased. For the copolymer p­(DOT_0.98_-*co*-DEVP_0.02_)*_n_* (Table 1, entry 4), which was formed with 5 equiv DEVP feed and 74% DOT conversion, just over half (51%) of the converted DEVP groups were located at the α end groups, meaning that chains carried, on average, less than one internal DEVP unit, especially when considering that some chains would have still been initiated by AIBN directly (Figure 8, spectrum C). For the copolymer made from the lowest DEVP feed (DOT–DEVP 50:1), p­(DOT_0.994_-*co*-DEVP_0.006_)*_n_* (Table 1, entry 7), 71% of incorporated DEVP was located at the α end groups, with the rest of the chains having a 99.8 mol % DOT content and roughly three in five chains carrying no internal DEVP groups and being AIBN–DEVP-initiated DOT homopolymers.

**Figure 8:**
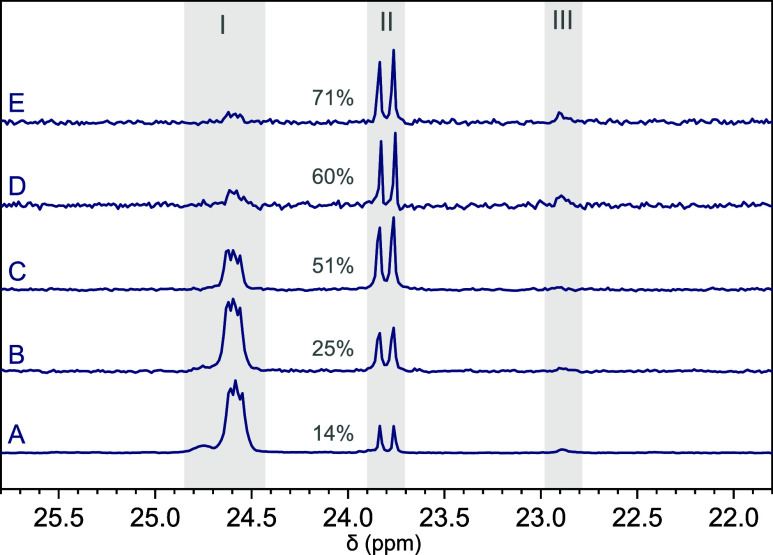
^31^P {^1^H} NMR spectra of DOT–DEVP copolymers made with [DOT]_0_:[AIBN]_0_ = 50:1 with a decreasing [DEVP]_0_ of (A) 50 (Table 1, entry 1), (B) 25 (entry 2), (C) 5 (entry 4), (D) 3 (entry 5), and (E) 1 (entry 7), with the signals associated with internal DOT–DEVP–DOT units (I) and AIBN–DEVP–DOT α end groups (II) highlighted with normalized integrals. There was insufficient data to assign a weaker signal (III).

This data highlights the importance of DEVP in initiating the polymerization of DOT. The addition equilibrium of AIBN onto DOT can be presumed to be on the left, with the tertiary, resonance-stabilized AIBN-derived radical being an ill-suited initiator for DOT. Instead, the AIBN-based radical adds onto DEVP in an irreversible step. The specific advantage of the DOT–DEVP pairing is the preference of DEVP to cross-propagate, as highlighted by the estimated reactivity ratio of *r*_DEVP_ = 0.01 (leading to a single monomer DEVP insertion at the α end group), while DOT prefers homopolymerization (*r*_DOT_ = 17) (leading to low conversion of DEVP following initiation). As DEVP is consumed during initiation, a lower feed of DEVP results in less DEVP remaining for in-chain incorporation. With a lower DEVP feed, initiation becomes less efficient (as presumably the formation of an AIBN-DEVP radical becomes less likely), explaining the observed lower conversions at nearly constant molecular weight.

The NMR spectra gave no evidence of chain transfer to monomer, which would have produced phosphorus atoms coupling to POCH­(R)­(Me) methine groups, which were not visible. Given the low estimated chain transfer constant and the high limit of detection of NMR spectroscopy, the occurrence of chain transfer could not be ruled out. To obtain further evidence of the comonomer initiation theory, DOT (co)­polymerizations were conducted in the presence of other additives; see Table 2. A polymerization of 50 DOT and 1 AIBN in the presence of 5 equiv of diethyl methylphosphonate (featuring a phosphonate without vinyl group) gave a DOT homopolymer with an SEC-measured *M*_n_ = 13.8 kg/mol in 12% conversion (Table 2, entry 1); see Figure 9. The conversion was similar to the 13% obtained in the absence of any additive (Table S1, entry 13) but with a slightly lower molecular weight compared to *M*_n_ = 14.8 kg/mol without an additive. This data suggested the possible occurrence of chain transfer to phosphonate but indicated that these events were not responsible for increasing the conversion of DOT. On the other hand, a copolymerization under the same conditions containing 5 equiv of *N*-phenyl maleimide (a monomer known not to homopolymerize and prefer alternating copolymerization with DOT)^27^ led to 46% DOT conversion, 8% *N*-phenyl maleimide conversion and a copolymer with a 98 mol % DOT content and an SEC-measured *M*_n_ = 13.8 kg/mol, Table 2, entry 2. An analogous reaction using 5 equiv of styrene gave comparable results with 52% DOT conversion, 10% styrene conversion, and a product containing 98 mol % DOT with an SEC-measured *M*_n_ = 17.7 kg/mol, Table 2, entry 3. The case of styrene was unexpected given that our initial study showed no copolymerization at all of the DOT–styrene system.^17^ Herein, the data indicate the importance of a comonomer to initiate the polymerization, which increases the conversion. Both styrene and *N*-phenyl maleimide are, however, more reactive than DEVP, which leads to the incorporation of larger amounts of comonomer, making DEVP the ideal choice to prepare DOT “homo”polymers.

**Figure 9:**
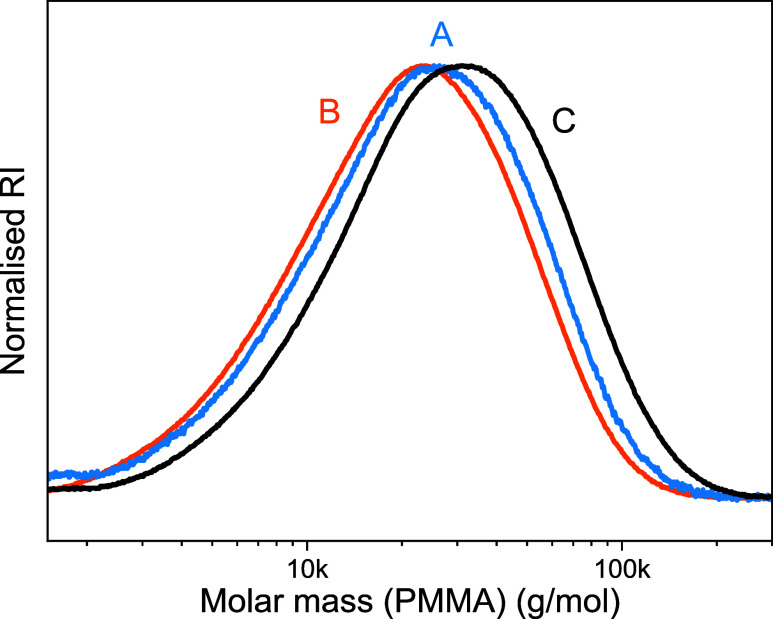
SEC curves of three (co)­polymers prepared under the same conditions: (A) DOT homopolymer prepared in the absence of additive (Table S1, entry 13): DOT conversion 13%, *M*_n_ = 14.8 kg/mol, *Đ* = 2.00; (B) DOT homopolymer prepared in the presence of 5 equiv of diethyl methylphosphonate (DEMP): DOT conversion 12%, *M*_n_ = 13.8 kg/mol, *Đ* = 1.91; and (C) DOT–DEVP copolymer prepared in the presence of 5 equiv of DEVP: DOT conversion 74%, *M*_n_ 19.3 = kg/mol, *Đ* = 1.91. The similar molecular weights and dispersities but drastically different conversions support the notion that the main role of the DEVP comonomer is to initiate the polymerization.

**Table 2:** Free Radical Copolymerization of DOT with Other Monomers in Anisole, Feed [DOT]:[Additive] = 50:5 with 1 AIBN

entry	product	additive	conversion DOT (%)	conversion additive (%)	*M*_n_ (*Đ*) ^*a*^
1	pDOT	diethyl methylphosphonate	12		13.8 (1.93)
2	p(DOT_0.98_-*co*-PhMI_0.02_)*_n_*	*N*-phenyl maleimide (PhMI)	46	8	13.8 (1.91)
3	p(DOT_0.98_-*co*-Sty_0.02_)*_n_*	styrene (Sty)	52	10	17.7 (1.83)

### Aminolytic and Thermal Degradation

With a series of DOT-rich copolymers in hand, we finally confirmed their degradation through aminolysis and through heating in the absence of solvent. Full degradation by aminolysis with ethylamine (1 M in THF) was confirmed on two examples, p­(DOT_0.92_-*co*-DEVP_0.08_)*_n_* and p­(DOT_0.97_-*co*-DEVP_0.03_)*_n_* (Table 1, entries 2 and 3, respectively) with SEC showing the complete disappearance of the polymer signals and formation of small-molecule fragments only, as to be expected for the absence of nondegradable DEVP–DEVP diads; see Figure 10.

**Figure 10:**
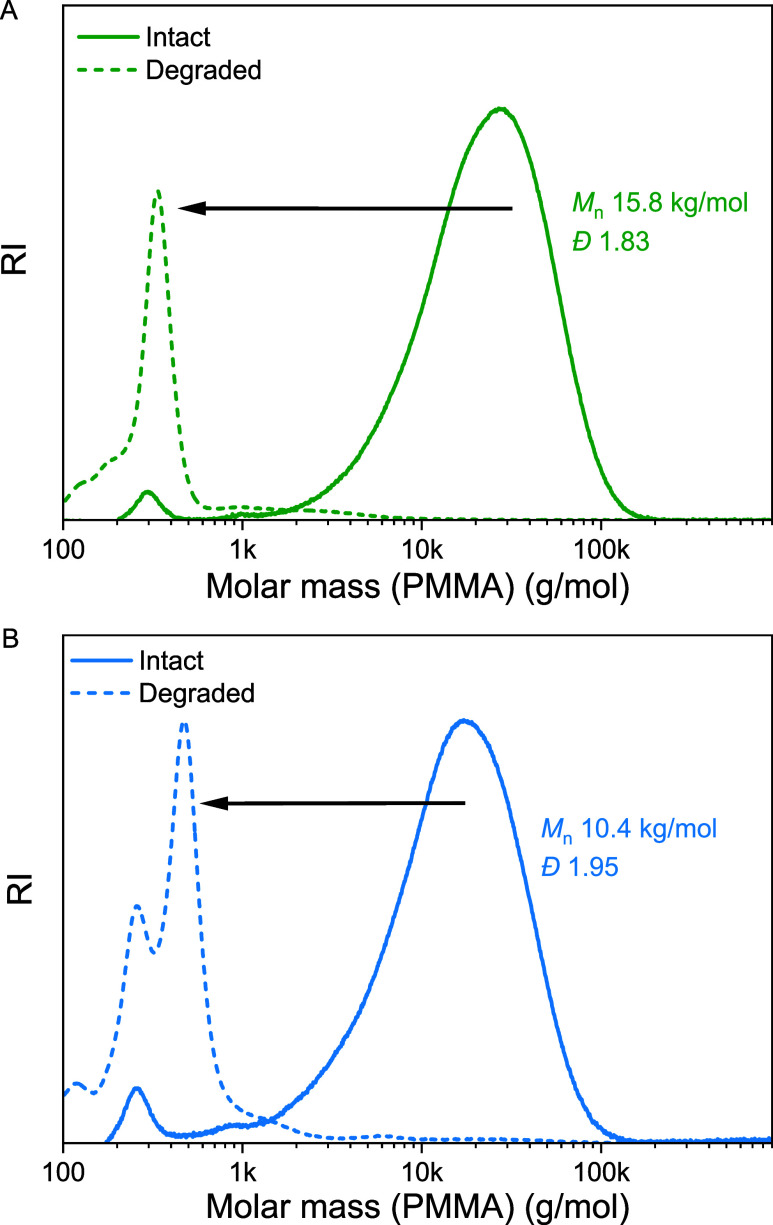
SEC traces of (A) p­(DOT_0.92_-*co*-DEVP_0.08_)*_n_* (Table 1, entry 2) and (B) p­(DOT_0.97_-*co*-DEVP_0.03_)*_n_* (Table 1, entry 3) intact (solid lines) and after treatment with 1 M ethylamine in THF (dashed lines).

We recently demonstrated the thermal degradation of (cationically made) pDOT homopolymers and showed the importance of the ω end group in initiating the unzipping to give DTO, the thioester isomer of DOT; see Scheme 1D. With a more labile ω end group, the thermal degradation of pDOT was reasonably fast, showing up to 35% DTO formation after heating to 140 °C in air for 20 h.^12^ Herein, the two copolymers with the highest (p­(DOT_0.82_-*co*-DEVP_0.18_)*_n_*, Table 1, entry 1) and lowest (p­(DOT_0.994_-*co*-DEVP_0.006_)*_n_*, Table 1, entry 7) DEVP contents were chosen and heated (without solvent) to 140 °C for 30 days in air. Withdrawn samples were analyzed by SEC and ^1^H NMR spectroscopy. Both copolymers showed a progressive reduction in molecular weight over time, as shown in Figure 11A,B. A plot of the SEC-measured molecular weight, normalized to the weight before heating, showed that the sample containing 18 mol % DEVP degraded considerably faster, reaching half of the original molecular weight after 1 day, while the copolymer containing 0.6 mol % DEVP required approximately 3 days to reach the equivalent molecular weight reduction, Figure 11C. NMR analysis of the withdrawn samples showed DTO to be the main small-molecule degradation product, Figure S13. The increasing formation of DTO with time was plotted with an inverted axis to allow a comparison with the SEC-observed loss of molecular weight (Figure 11C). If a polymer degrades by every chain unzipping directly into small molecules, the two curves can be expected to overlay, e.g., a 30% reduction in average molecular weight would result in a formation of 30% DTO. Our data indicated that the degradation of the DOT–DEVP copolymers did not follow this path, with the formation of DTO lagging behind the reduction of molecular weight and suggested that copolymers first degraded (presumably hydrolytically through atmospheric water) into oligomeric fragments. Cyclization into DTO was presumed to occur through backbiting of a terminal thiol; see Scheme S1. Surprisingly, the two DOT–DEVP copolymers showed different behavior. After 30 days of heating to 140 °C, the DEVP-poor species p­(DOT_0.994_-*co*-DEVP_0.006_)*_n_* (triangles in Figure 11C) had degraded to less than 10% of its original molecular weight, with NMR showing 7 mol % of DTO next to residual (undegraded uncyclized) oligomeric fragments. The DEVP-rich sample, p­(DOT_0.82_-*co*-DEVP_0.18_)*_n_*, on the other hand (squares in Figure 11C) degraded to a similarly small percentage of its original molecular weight but with the formation of 83% DTO (this figure was determined by comparison of the characteristic DTO methylene NMR resonances with the sum of the aromatic signals and thus ignored the DEVP content). It was assumed that DTO formation was not possible from DOT-DEVP diads; see Scheme S1. This more efficient formation of the small-molecule building block DTO was surprising. In an attempt to learn about the effect of DEVP repeat units during heating, a sample of the DEVP homopolymer (Table 1, entry 12) was heated to 140 °C for 24 h. The sample turned black and became insoluble in common solvents, preventing NMR analysis. Presumably, thermal decomposition products of DEVP repeat units^48^ catalyzed the cyclization of DOT repeat units into DTO. The observed thermal degradation data is in stark contrast to the thermogravimetric analysis (TGA) discussed above (Figure S6) which showed stability of DOT–DEVP copolymers until 300 °C. Notably, there are two important differences between the experiments: (i) TGA was performed with a heating rate of 10 °C, while heating to 140 °C was carried out for 30 days; (ii) TGA only detects decomposition through the loss of volatile substances; any fragmentation into nonvolatile oligomers is not detected. Given that applications may involve prolonged heating and that a reduction in molecular weight may affect material properties, it is therefore important for degradable polymers to be characterized by not only TGA but also SEC.

**Figure 11:**
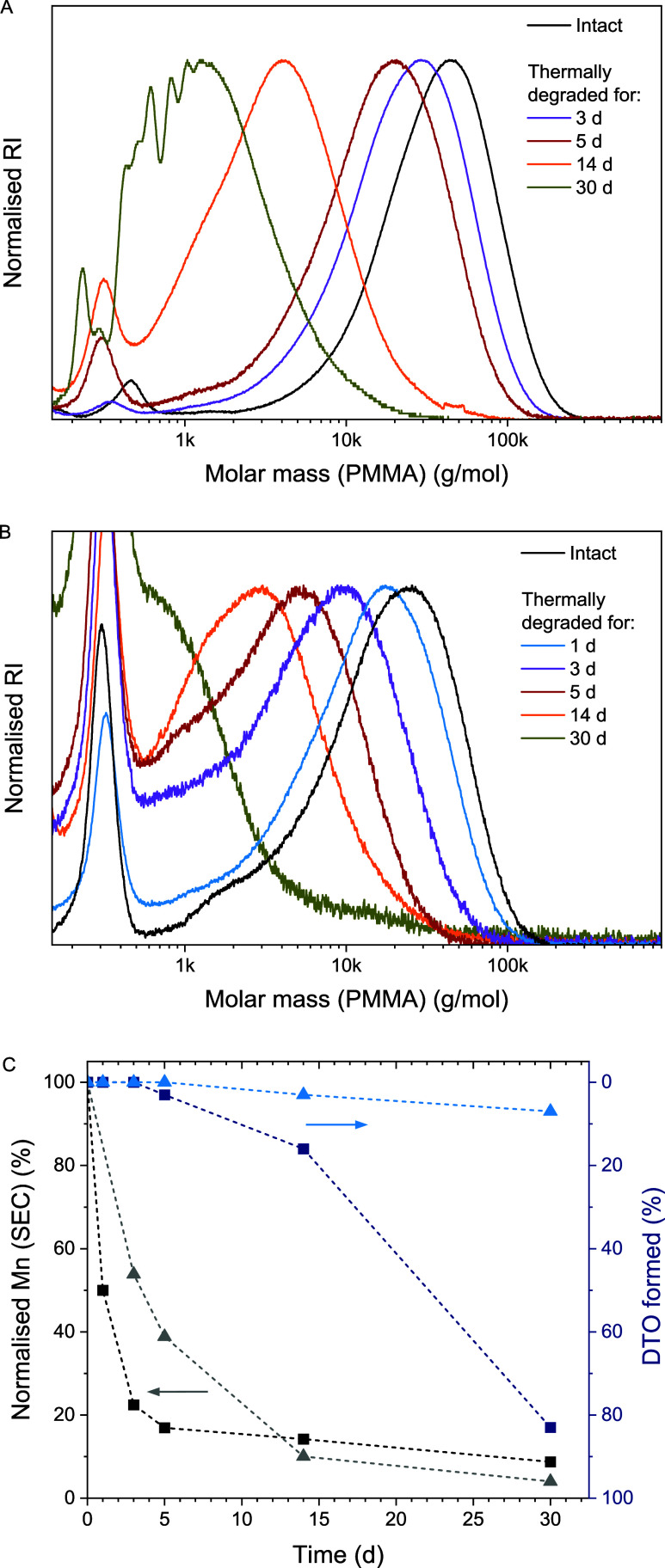
Heating of dry DOT–DEVP copolymers to 140 °C for 30 d: SEC curves over time for (A) p­(DOT_0.994_-*co*-DEVP_0.006_)*_n_* (Table 1, entry 7) and (B) p­(DOT_0.82_-*co*-DEVP_0.18_)*_n_* (Table 1, entry 1); (C) evolution of SEC-measured molecular weight (normalized to initial value, left axis, black/gray data) and percentage of DTO formed from NMR analysis (right axis, blue data) for p­(DOT_0.994_-*co*-DEVP_0.006_)*_n_* (Table 1, entry 7) (triangles) and p­(DOT_0.82_-*co*-DEVP_0.08_)*_n_* (Table 1, entry 1) (squares). The right axis is plotted in reverse to show degradation events in a downward direction and allow comparison with the SEC data.

## Conclusions

A distinguishing feature of thiocarbonyl monomers is that radical additions are reversible. The equilibrium for the addition of an initiator-derived radical lying on the left can therefore inhibit effective initiation, as appears to be the case for the AIBN-initiated homopolymerization of DOT. Our study showed that the addition of a secondary more-activated monomer (styrene, *N*-phenyl maleimide, DEVP) presents a possible workaround because the addition of the AIBN-derived radical onto these is irreversible and the monomer-based secondary radical shows a great affinity for DOT. In this context, DEVP proved advantageous because of its low propensity to homopolymerize. Uniquely, ^31^P NMR spectroscopy could be used to elucidate the chemical surroundings of the DEVP repeat units to a distance of up to 5 bonds and to quantify the percentage of internal DEVP units. Thus, DOT-rich polymers (to the extent of being DEVP-initiated DOT homopolymers) with tunable lengths were prepared with high global conversions. The usage of initiators that produce secondary (or more reactive) radicals is anticipated to lead to DOT homopolymers in the absence of a comonomer. The incidental observation of the drastic influence of DEVP repeat units on the outcome of thermal degradation presents an unexplored avenue to tune degradation toward producing building blocks suitable for circularity.

## Supplementary Material



## Notes

DOT homopolymerization experiments; DOT ^1^H NMR data in various solvents; estimation of reactivity ratios, NMR, FT-IR, and DSC; SEC analysis of DOT–DEVP copolymers; and thermal degradation (PDF)
