# Supplementary material for: Single-Unit Comonomer Insertion Initiates Radical Polymerization of Thionolactone to Give Chemically and Thermally Degradable Polythioesters
Source: Macromolecules. 2025 Sep 5;58(18):9617–28. doi: 10.1021/acs.macromol.5c01418 (PMC12461944; doi:10.1021/acs.macromol.5c01418)
Supplement: Supplementary file 1 [file ma5c01418_si_001.pdf]

— : — **Supporting Information** — : —

Single Unit Comonomer Insertion Initiates Radical Polymerization of Thionolactone to Give Chemically and Thermally Degradable Polythioesters

*Touseef Kazmi, Kyle Hepburn, Qamar Nisa, Swarnali Neogi, Nathaniel M. Bingham, Peter J. Roth**

School of Chemistry and Chemical Engineering, University of Surrey, Guildford, Surrey, GU2 7XH, UK; * corresponding author email address p.roth@surrey.ac.uk

Contents

1. Homopolymerizations of DOT 2
2. ^{1}H NMR spectra of DOT in various solvents 3
3. Reactivity ratios DOT–DEVP 5
4. Analysis of DOT–DEVP Copolymers 6
5. Degradation of Copolymers 11
6. References 12

1. Homopolymerizations of DOT

Table S1. Homopolymerizations of DOT

Entry	Initiator (I)	$[DOT]_0/[I]_0$	Solvent [a]	Temp. (°C)	Reaction time (h)	Conversion (%)	M_n (kg/mol) ($Đ$) [b]
1	AIBN	50	MeCN	70	19	0	
2	AIBN	100	MeCN-d_3	70	19	2	
3	AIBN	50	DMSO-d_6	70	19	2	
4	AIBN	100	DMSO-d_6	70	19	3	
5	AIBN	50	DMF	70	19	12	
6	AIBN	50	acetic acid	70	19	10	
7	AIBN	50	ethyl acetate	70	120	7	
8	AIBN	50	toluene	70	19	31	15.0 (2.06)
9	AIBN	50	toluene	70	19	12	
10	AIBN	50	toluene-d_8	70	19	13	
11	AIBN	50	toluene–octane (1:1)	70	19	12	
12	AIBN	50	toluene–ethanol (4:1)	70	19	7	
13	AIBN	50	anisole	70	19	13	14.8 (2.00)
14	AIBN	50	anisole [c]	70	19	3	4.7 (1.30)
15	AIBN	50	anisole	70	170	6	5.5(1.44)
16	lauroyl peroxide	100	DMSO-d_6	70	19	1	
17	benzoyl peroxide	100	DMSO-d_6	70	19	1	
18	di-*tert* butyl peroxide	50	anisole	120	19	3	6.8 (2.31)
19	di-*tert*-butyl peroxide	100	anisole	120	19	0	
20	AIBN [d]	100	MeCN-d_3	70	19	2	
21	AIBN [e]	100	MeCN-d_3	70	19	0	

[a] [DOT] = 0.15 M unless otherwise noted; [b] Determined by SEC analysis; [c] [DOT] = 0.6 M; [d] AIBN (0.25 eq) and macro-RAFT agent poly[oligo(ethylene glycol) methyl ether acrylate]$_{24}$ (1 eq) were used; [e] AIBN (0.25 eq) and macro-RAFT agent poly[oligo(ethylene glycol) methyl ether acrylate]$_{99}$ (1 eq) were used

2. ^{1}H NMR spectra of DOT in various solvents

Table S2. ^{1}H NMR spectra run on DOT solutions in various deuterated and non-deuterated solvents: details of CH_2 group signals [a]

Entry	Solvent [a]	CH_2 Multiplicity	δ_1 (ppm) [b]	δ_2 (ppm) [b]	$\Delta\delta$ (ppm)	^2J (Hz)
1	DMSO-d_6	2×d	5.45	5.14	0.31	11.7
2	Benzene-d_6	2×s [c]	4.69	4.68	0.01	—
3	$CDCl_3$	2×d	5.24	5.16	0.08	11.4
4	Diethyl ether	2×s [c]	5.24	5.23	0.01	—
5	Acetonitrile	2×d	5.45	5.29	0.16	12.0
6	Acetic acid	2×d	5.28	5.16	0.12	11.8
7	Acetone	2×d	5.45	5.27	0.18	11.6
8	Dioxane	2×d	5.38	5.26	0.12	11.2
9	DMF	2×d	5.59	5.37	0.22	11.2
10	Ethyl acetate	2×d	5.40	5.24	0.16	11.0
11	Methyl acrylate	2×d	5.26	5.12	0.14	11.4
12	THF	2×d	5.33	5.21	0.12	11.2
13	Toluene-d_8	2×s [c]	4.72	4.71	0.01	—
14	Toluene–hexane (1:1)	2×s [c]	4.73	4.72	0.01	—
15	Toluene-d_8–octane (1:1)	s	4.56	—	0	—
16	Anisole	s	5.07	—	0	—

[a] Non-deuterated solvents were used as long as their signals did not overlap with the DOT CH_2 signal; [b] Samples in non-deuterated solvents were run in "no solvent" mode and reported chemical shifts are based on calibration of the residual solvent signals to their measured chemical shift in $CDCl_3$; [c] due to strong roofing, two adjacent doublets appeared as two singlets

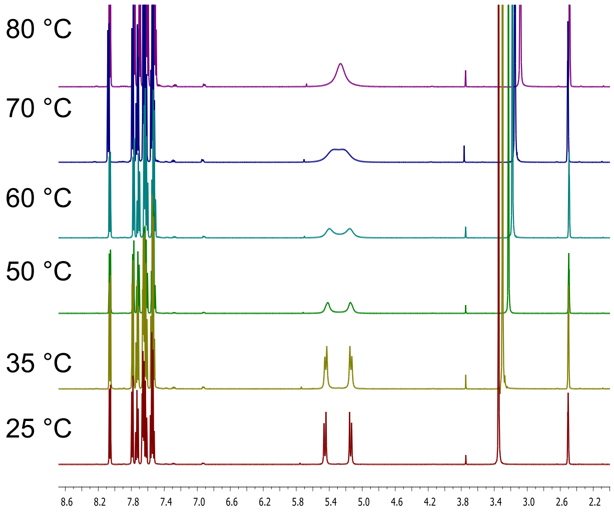

Figure S1. Variable temperature ^{1}H NMR spectra of DOT run in DMSO-d_6 at temperatures from 25–80 °C. The converging of the two doublets shows the barrier to the inversion of DOT which makes the two CH_2 protons equivalent on the NMR timescale.

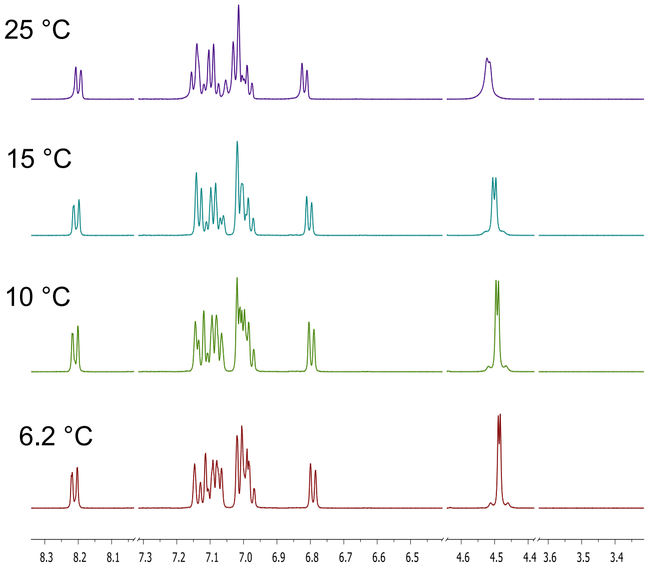

Figure S2. Variable temperature ^{1}H NMR spectra of DOT run in toluene-d_8 at different temperatures from 6.2–25 ºC. At room temperature, the spectrum shows a broad doublet which starts to split into two doublets (with strong roofing) upon cooling to 6.2 °C (the lowest temperature available on our NMR spectrometer). A comparison with the measurements in DMSO (Figure S1) shows that the barrier to the inversion depends on the solvent.

3. Reactivity ratios DOT–DEVP

The expected conversion was calculated numerically by starting with a batch of 100 monomers (divided 50:50 into DOT and DEVP based on their feed ratio). The global conversion was divided into 100 steps, at each of which the comonomers are consumed based on the Mayo-Lewis equation (1) using their instantaneous comonomer mole fractions, f_{DOT} and f_{DEVP}. The sum of comonomers consumed at each step was $F_{DOT}^{inst} + F_{DEVP}^{inst} = 1$. The mole fractions for the next global consumption step were calculated after subtracting the consumed comonomer amount from the remaining feed. When a comonomer was fully consumed, its amount was set to zero and the iteration was allowed to continue for the remaining comonomer to be consumed. The experimental data was fitted by minimising the mean squared errors.

$$F_{DOT}^{inst} = \frac{r_{DOT} f_{DOT}^2 + f_{DOT} f_{DEVP}}{r_{DOT} f_{DOT}^2 + 2 f_{DOT} f_{DEVP} + r_{DEVP} f_{DEVP}^2} \qquad (1)$$

where F_{DOT}^{inst} = mole fraction of instantaneously incorporated DOT,

f_{DOT} and f_{DEVP} = instantaneous mole fractions of residual DOT and DEVP

r_{DOT} and r_{DEVP} = reactivity ratios of DOT and DEVP

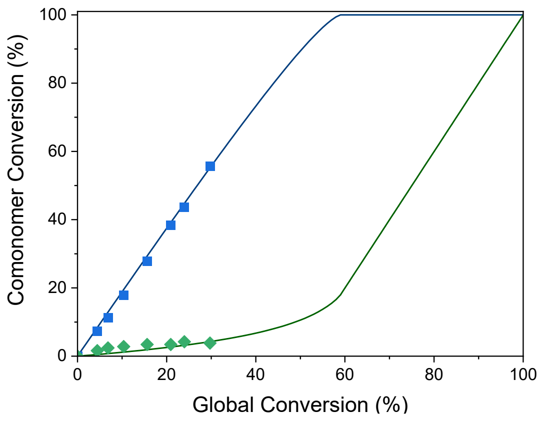

Figure S3. Calculated conversion of DOT (blue line) and DEVP (green line) plotted versus global conversion with experimental data (DOT: blue squares, DEVP: green diamonds) for the copolymerization of a 50 DOT–50 DEVP feed (Table 2, entry 8). Data points are available only for the first section as higher conversions were not achieved. The fitted lines use the parameters r_{DOT} = 17 and r_{DEVP} = 0.01. This method provides a rough estimation only as it is based on a single copolymerization and relies on the determination of low conversions by NMR integration.[1] Nonetheless, the significantly higher reactivity of DOT compared to DEVP is reflected in the estimated values.

4. Analysis of DOT–DEVP Copolymers

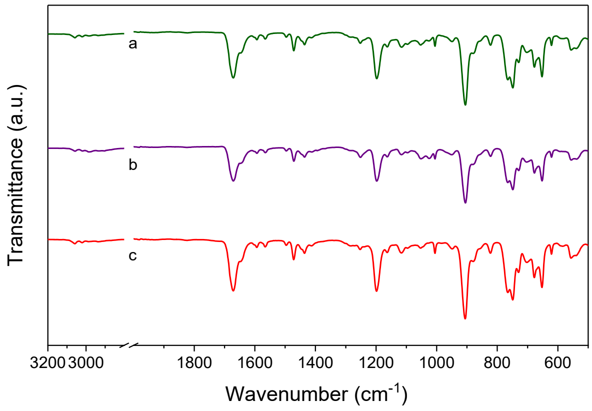

Figure S4. FT-IR spectra of (a) p(DOT$_{0.92}$-*co*-DEVP$_{0.08}$)$_n$ (Table 2, entry 2), (b) p(DOT$_{0.98}$-*co*-DEVP$_{0.02}$)$_n$ (Table 2, entry 4), and (c) pDOT homopolymer (Table 1, entry 13) showing virtually identical spectra due to the small DEVP content.

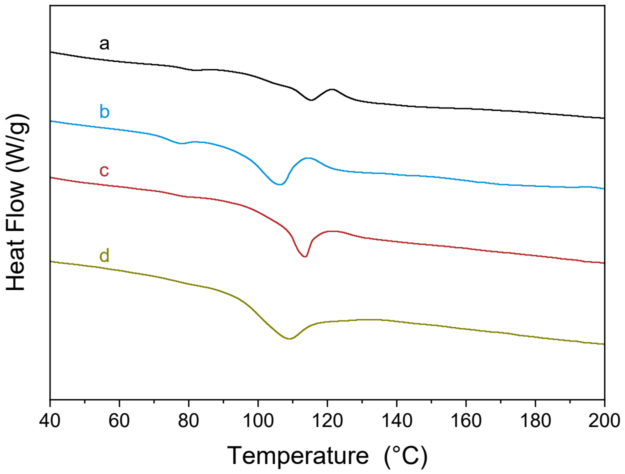

Figure S5. Differential scanning calorimetry (second heating curves) of (a) p(DOT$_{0.92}$-*co*-DEVP$_{0.08}$)$_n$ (Table 2, entry 2), (b) p(DOT$_{0.98}$-*co*-DEVP$_{0.02}$)$_n$ (Table 2, entry 4), (c) p(DOT$_{0.99}$-*co*-DEVP$_{0.01}$)$_n$ (Table 2, entry 6), and (d) pDOT homopolymer (Table 1, entry 8)

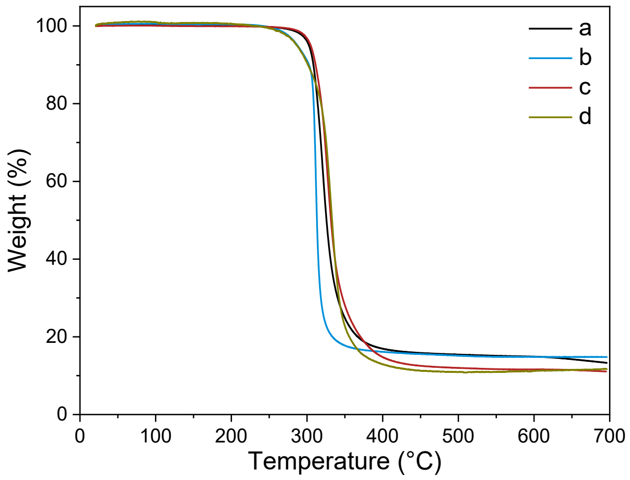

Figure S6. Thermogravimetric analysis of (a) p(DOT$_{0.92}$-*co*-DEVP$_{0.08}$)$_n$ (Table 2, entry 2), (b) p(DOT$_{0.98}$-*co*-DEVP$_{0.02}$)$_n$ (Table 2, entry 4), (c) p(DOT$_{0.99}$-*co*-DEVP$_{0.01}$)$_n$ (Table 2, entry 6), and (d) pDOT homopolymer (Table 1, entry 8) showing that the presence of DEVP repeat units did not influence the thermal stability.

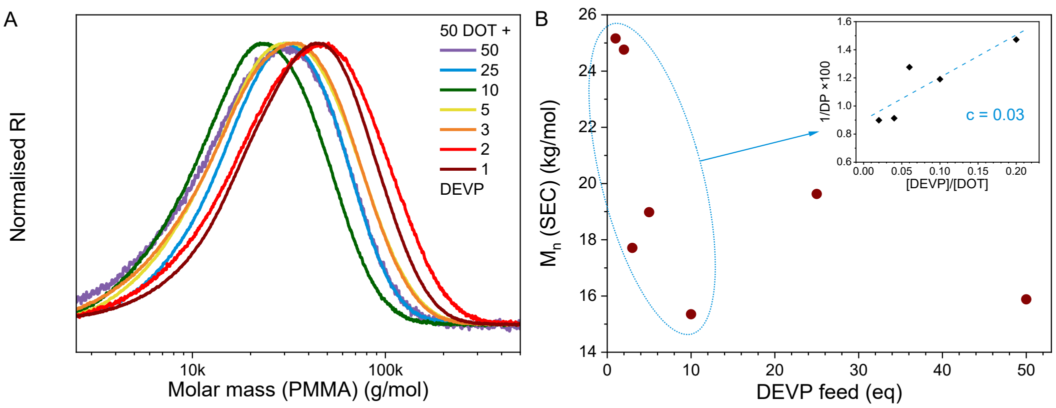

Figure S7. (A) SEC curves for a series of copolymers with varying feed of DEVP; (B) the plot of molecular weights versus DEVP feed showed a decrease in SEC-measured molecular weight with an increasing DEVP feed for the first 5 samples only. Plotting the inverse of the degree of polymerization (using the PMMA-equivalent SEC-measured molar masses as estimation) versus the [DEVP]$_0$/[DOT]$_0$ ratio (inset) gave a chain transfer constant, c, of 0.03.

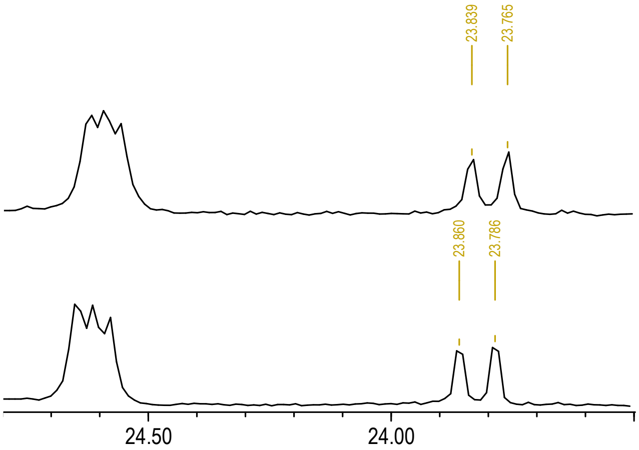

Figure S8. Stacked $^{31}P\{^{1}H\}$ NMR spectra of p(DOT$_{0.92}$-*co*-DEVP$_{0.08}$)$_n$ (Table 2, entry 2) on a 400 MHz (top) and a 500 MHz (bottom) spectrometer.

Table S3. Peak separation in Hz and ppb of the apparent doublet in Figure S7 indicates that the splitting is not due to coupling but different ^{31}P environments.

Spectrometer	Frequency for ^{31}P	Peak Difference	Apparent coupling constant [a]
	MHz	ppb	Hz
400 NMR	161.9	74	12
500 NMR	202.4	74	15

[a] peak difference × frequency

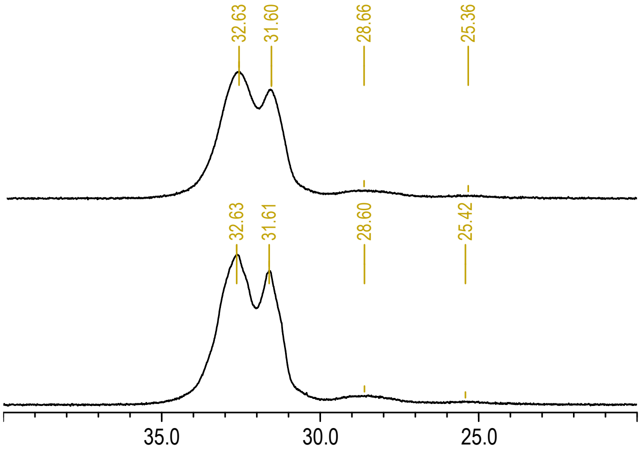

Figure S9. ^{31}P NMR (top) and hydrogen-decoupled $^{31}P\{^{1}H\}$ NMR (bottom) stacked spectra of pDEVP (Table 2 entry 12).

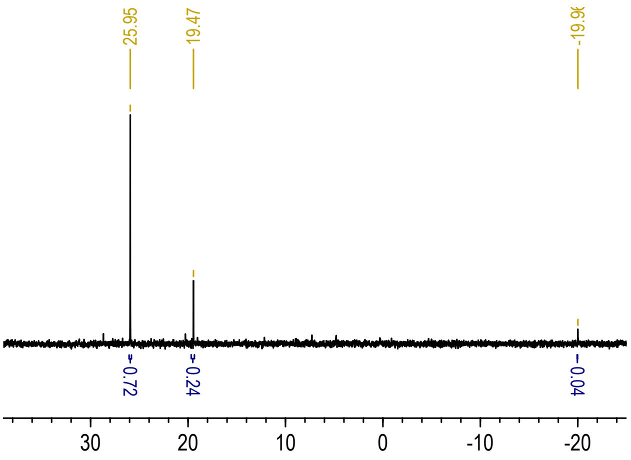

Figure S10. Larger section of the $^{31}P\{^{1}H\}$ NMR spectrum of the ethylamine-degraded copolymer p(DOT$_{0.92}$-*co*-DEVP$_{0.08}$)$_n$ (Table 2, entry 2) showing an additional minor signal at −20 ppm.

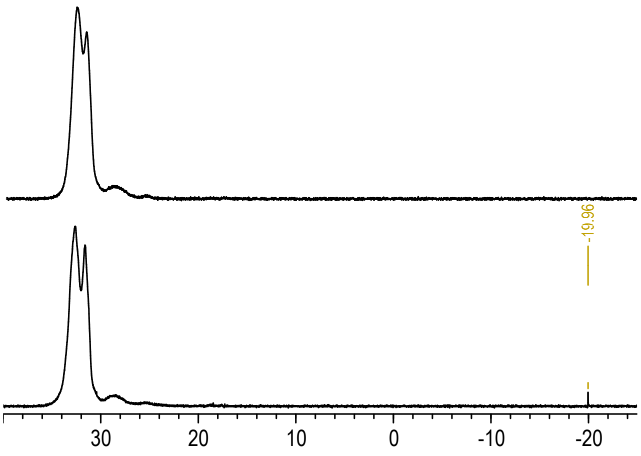

Figure S11. Stacked $^{31}P\{^{1}H\}$ NMR spectra of DEVP homopolymer (Table 2, entry 12) (top) and after treatment with ethylamine (bottom), showing no 'degradation' apart from a minor signal at −20 ppm, assigned to a phosphoramide formed by substitution of ethanol on phosphorous.

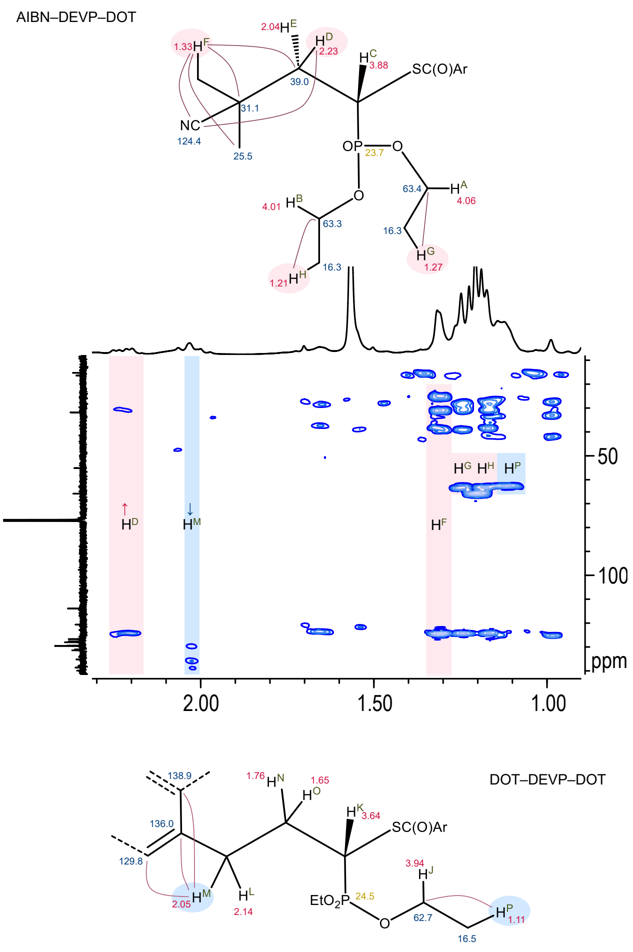

Figure S12. ^{1}H–^{13}C HMBC spectrum (middle) with structures distinguished by their ^{31}P–^{1}H interactions (see Figure 7). Interactions associated with AIBN–DEVP–DOT α end groups (red, top structure) and DOT–DEVP–DOT triads (blue, bottom structure) are highlighted.

5. Degradation of Copolymers

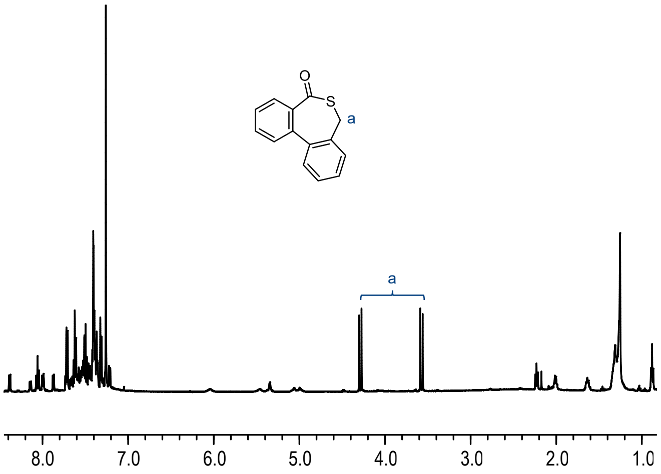

Figure S13. ^{1}H NMR spectrum of p(DOT$_{0.82}$-*co*-DEVP$_{0.18}$)$_n$ (Table 2 entry 1) after heating to 140 °C for 30 days showing the characteristic two doublets of DTO.[2]

Scheme S1. Proposed mechanism for the thermal degradation of DOT–DEVP copolymers. Following the random hydrolysis of an internal thioester, backbiting gives the cyclic thioester product, DTO. When a DEVP repeat unit is reached (bottom left), cyclization to a 9-membered ring is not expected to occur but the phosphonate thiol may undergo onward reactions under the conditions.

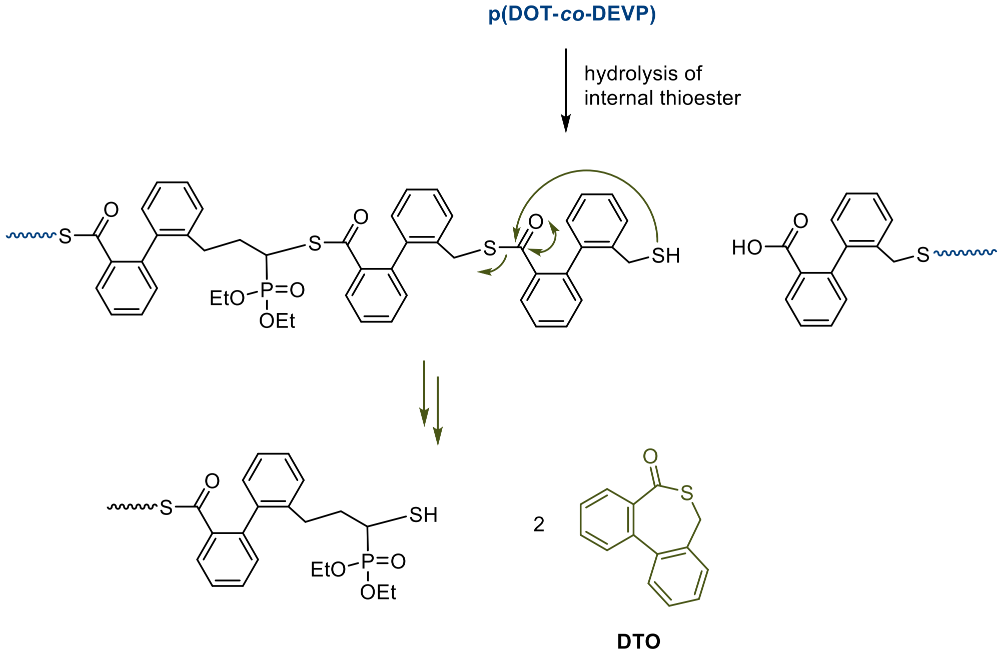

6. References

(1) Autzen, A. A. A.; Beuermann, S.; Drache, M.; Fellows, C. M.; Harrisson, S.; van Herk, A. M.; Hutchinson, R. A.; Kajiwara, A.; Keddie, D. J.; Klumperman, B.; et al. IUPAC recommended experimental methods and data evaluation procedures for the determination of radical copolymerization reactivity ratios from composition data. *Polymer Chemistry* **2024**, *15* (18), 1851-1861, 10.1039/D4PY00270A. DOI: 10.1039/D4PY00270A.

(2) Neogi, S.; un Nisa, Q.; Abu Bakar, R.; Bingham, N. M.; Roth, P. J. Ambient cationic ring-opening polymerization of Dibenzo[c,e]oxepine-5(7H)-thione (DOT): Thermal and nucleophile-initiated depolymerization. *European Polymer Journal* **2024**, *219*, Article. DOI: 10.1016/j.eurpolymj.2024.113390 Scopus.
